# Health Tract No. 313

**Published:** 1868-03

**Authors:** 


HEALTH TRACT No. 313.

### SALT OF THE EARTH.

Without religion, this planet would not contain one solitary human inhabitant in all the ages. The salt of the sea preserves it from corruption and unbearable noisomeness; it is the salt in the human body, which prevents physical decay. It is the moral salt, the preservative influences of the christian religion, which upholds social existence, which sustains all civilized governments and prevents the extinction of nationalities; and this too is the preservative influence in the individual, which saves him from bestiality and crime and degradation; any man without it becomes a savage; it is this which throws around woman the halo of her holiness and her purity and her social exaltation; hence it is, that the enemies of religion are the vipers of society; they poison and corrupt and destroy all social influences for good, and wherever they habitate together, crime and beastliness, in their most degrading, disgusting and most horrid forms reign rampant, society has no guarantees, decency is outraged, law has no power and virtue is extinct.

These are not the vagaries of an excited immagination, for not only has the Divine Master declared of his followers "Ye are the salt of the earth," but actual facts multiply in these later ages, demonstrating the truth of the principle; one may be stated as a sample of multitudes of others.

Some years ago a town was founded by a set of German infidels, one article of the organization was, that no house of religious worship should ever be erected on the spot. A few years later, a terrible calamity befel the place, and as if the curse of Heaven attached itself to the illfated town, a more recent incident has occurred which will shock humanity wherever it is narrated. Three strangers stopped at the public house in the village within a year, it was towards evening, and they expected to resume their journey the next morning. Without any atequate cause, they were set upon and beaten in the most savage manner; one of them not being quite dead, yet in a dying condition, was taken up and hung; these statements are made from memory. The judicial record brings up the history to this date, ten months after the occurrence in the language of an intelligent gentleman on the spot;

"Well might the Judge remark, when commenting upon the conduct of the jury, that he had antiticipated the strength of these influences in New Ulm, and to avoid them as far as possible had removed the court forty miles from there, but that it was evident it had not been removed far enough to get beyond their reach. That when three men had lost their lives by violence, on the same day, in the same village; that when there were 150 witnesses to the killing of two of them, and they (the Grand Jury) had had a selection from a the whole number, and as many as they could hear the testmony of in six days, and could yet return upon their oaths, that no offence had been commintted, or if any offence, that there was not evidence before them as to who were the guilty parties, presented a mournful and unprecedentend state of things, although the jury would never by their action convince either Court, or people that no offence had been committed or that there was no evidence to charge anybody with its commission.

And but for the controlling influences of the principles of the Christian religion, New-York, with all its wealth and grandeur and intellect would become in a short time the same Pandemonium as New-Ulm of Minnesota.

